# Observation of the Gut Microbiota Profile in C57BL/6 Mice Induced by *Plasmodium berghei* ANKA Infection

**DOI:** 10.3389/fcimb.2021.680383

**Published:** 2021-10-28

**Authors:** Wei Guan, Shuguo Yang, Yanqing Zhao, Weijia Cheng, Xiaonan Song, Yi Yao, Yiting Xie, Weixing Du, Jian Li

**Affiliations:** ^1^ Department of Human Parasitology, School of Basic Medicine, Hubei University of Medicine, Shiyan, China; ^2^ Department of Infectious Diseases, Renmin Hospital, Hubei University of Medicine, Shiyan, China

**Keywords:** *Plasmodium berghei*, cerebral malaria, gut microbiota, 16S rRNA, infection

## Abstract

The genus of *Plasmodium* parasites can cause malaria, which is a prevalent infectious disease worldwide, especially in tropical and subtropical regions. C57BL/6 mice infected with *P. berghei* ANKA (PbA) will suffer from experimental cerebral malaria (ECM). However, the gut microbiota in C57BL/6 mice has rarely been investigated, especially regarding changes in the intestinal environment caused by infectious parasites. *P. berghei* ANKA-infected (PbA group) and uninfected C57BL/6 (Ctrl group) mice were used in this study. C57BL/6 mice were infected with PbA *via* intraperitoneal injection of 1 × 10^6^ infected red blood cells. Fecal samples of two groups were collected. The microbiota of feces obtained from both uninfected and infected mice was characterized by targeting the V4 region of the 16S rRNA through the Illumina MiSeq platform. The variations in the total gut microbiota composition were determined based on alpha and beta diversity analyses of 16S rRNA sequencing. The raw sequences from all samples were generated and clustered using ≥ 97% sequence identity into many microbial operational taxonomic units (OTUs). The typical microbiota composition in the gut was dominated by *Bacteroidetes*, *Firmicutes*, *Proteobacteria*, and *Verrucomicrobia* at the phylum level. *Bacteroidetes* and *Verrucomicrobia* were considerably decreased after PbA infection compared with the control group (Ctrl), while *Firmicutes* and *Proteobacteria* were increased substantially after PbA infection compared with Ctrl. The alpha diversity index showed that the observed OTU number was increased in the PbA group compared with the Ctrl group. Moreover, the discreteness of the beta diversity revealed that the PbA group samples had a higher number of OTUs than the Ctrl group. LEfSe analysis revealed that several potential bacterial biomarkers were clearly related to the PbA-infected mice at the phylogenetic level. Several bacterial genera, such as *Acinetobacter*, *Lactobacillus*, and *Lachnospiraceae_NK4A136_group*, were overrepresented in the PbA*-*infected fecal microbiota. Meanwhile, a method similar to gene coexpression network construction was used to generate the OTU co-abundance units. These results indicated that *P. berghei* ANKA infection could alter the gut microbiota composition of C57BL/6 mice. In addition, potential biomarkers should offer insight into malaria pathogenesis and antimalarial drug and malaria vaccine studies.

## Introduction

Malaria is the most prevalent infectious disease in tropical and subtropical regions, and it is caused by parasites of the genus *Plasmodium* (De et al., 2016). Malaria is a severe life-threatening disease with approximately 229 million clinical cases and 409,000 deaths in 2019 (WHO, 2020). Malaria has many economic burdens in addition to its health risks (Waide et al., 2020). Human malaria is caused by infection with four different *Plasmodium* parasites. *Plasmodium falciparum* causes cerebral malaria. In addition, gastrointestinal symptoms such as abdominal pain, diarrhea, and vomiting, develop in patients with *P. falciparum* malaria (Prasad and Virk, 1993). *P. falciparum* is the most lethal species, and it can cause acute encephalopathy, called human cerebral malaria (HCM) (Ghazanfari et al., 2018).

While there is still much to understand before gut microbiota modulation becomes a viable and optimal treatment to prevent severe malaria, recent evidence in both rodent models and human studies has pointed to the gut microbiota composition as a factor in disease progression. C57BL/6 mice with ECM caused by infection with PbA share several similarities to HCM by infection with *P. falciparum*. Namely, the C57BL/6 mice infected with PbA are a representative model of ECM for studying the mechanisms of human *Plasmodium* infections (Hunt et al., 2010).

Malaria infections affect the intestinal tract, and changes in the intestinal environment appear to influence malaria pathogenesis. The human body supports trillions of microbes, and most microbes inhabit the gastrointestinal tract (Gill et al., 2006). Sequencing technology has been applied to study the role of the microbiota in the relationships of health and disease in many kinds of research (Yooseph et al., 2015). Previous studies have shown that malaria can cause alterations in the host immune system (Long and Zavala, 2017), intestinal pathological changes (Taniguchi et al., 2015), and other effects, which suggest that the intestinal microbiota may modify the pathogenesis of infectious diseases and that the variability in the gut microbiota influences systemic immune responses. Yilmaz et al. found that mice colonized by *E. coli* O86:B7 induced specific antibodies that cross-reacted with *Plasmodium* spp. (Yilmaz et al., 2014). In addition, previous studies demonstrated that variations in the gut microbiota were associated with the onset of several diseases, such as enteric infections, diabetes mellitus, cardiovascular disease, colorectal cancer, reactive airway disease, and mood disorders (Wang et al., 2011; Arrieta et al., 2015; Komaroff, 2017; Qu et al., 2017; Dejea et al., 2018).

Moreover, Quigley’s study suggested that gut bacteria could impact the brain, and the microbiota has become a potential diagnostic and therapeutic target in cerebral disorders such as Parkinson’s disease, Alzheimer’s disease (AD), autism, stroke, depression, and so on (Quigley, 2017). Taniguchi et al. used C57BL/6 and BALB/c mice infected with PbA to show that alterations in the gut microbiota were related to intestinal pathological changes (Taniguchi et al., 2015). Fan et al. found that the gut microbiota composition in C57BL/6 mice was reconstructed after infection by the elimination of blood-stage PbA (Fan et al., 2019). Although these studies suggest a possible relationship between gut bacteria and malaria, the validity of the relationship has uncertainty due to the absence of direct evidence. Changes in the microbiota due to PbA infection can cause alterations in the immune status, metabolism, and so on, which can affect the malaria disease at the same time. Therefore, it will be a long process to study the mechanism of the relationship between malaria and changes in gut microbiota. Denny et al. showed that there was an increase in the proinflammatory cells in the lamina propria and changes in the cecal metabolites in C57BL/6 mice with differing susceptibility to *P. yoelii* 17XNL (Denny et al., 2019). However, some *Plasmodium* parasites appear to have developed resistance to available antimalarials, and there is no effective or long-lasting vaccine against malaria. The relationship between gut microbiota and human health and disease has been studied widely in recent years. Therefore gut microbiota modulation may be a potential treatment for severe malaria.

Recent studies have revealed that gut microbiota could be altered in various diseases, including neurodegenerative diseases, which led us to hypothesize that malaria infections may affect the gut microbiota. C57BL/6 mice with PbA infection is a severe ECM model to explore the possible role of PbA in influencing the gut microbiota of infected C57BL/6 mice. Therefore, the goals of this study were (1) to analyze the gut microbiota composition obtained from the feces of PbA-infected and uninfected mice separately by targeting the V4 region of the 16S rRNA through the Illumina MiSeq platform and (2) to identify biomarkers to offer insight into research into cerebral malaria pathogenesis, antimalarial drugs, and malaria vaccine studies.

## Materials and Methods

### Ethics Statement

The animals were maintained and used according to the Regulations for the Administration of Affairs Connecting Experimental Animals in China and the international research animal use guidelines. The protocol was approved by the Institutional Animal Care and Use Committee of the Hubei University of Medicine (HBMU-S20160414), and the mice were housed in the Animal Center of Collegial Laboratory.

### Mice and Infection With *Plasmodium berghei* ANKA

Six- to eight-week-old female C57BL/6 mice (22–30 g weight) were purchased from HNSJA Co., Ltd., Changsha, China, and maintained under specific pathogen-free conditions. The mice were fed an ultraviolet illuminated diet and pure water, which maintained their appropriate standards of living and feeding experimental conditions (25 ± 3°C) (Zhao et al., 2016). All mice were acclimatized to the living environment one week before the experiment. Twenty-six female C57BL/6 mice were randomly divided into two groups: PbA-infected mice (PbA) and PbA-uninfected mice (Ctrl).

The pathogen parasite PbA strain maintained in C57BL/6 mouse blood cells was obtained from Professor Wenyue Xu (Third Military Medical University). For infecting the donor C57BL/6 mice, a cryogenic vial containing frozen iRBCs was removed from liquid nitrogen and rapidly resuscitated in a 37°C water bath. The donor mouse was intraperitoneally infected with 100 μl of resuscitated iRBCs. After a one-week acclimation period in the experimental environment (Oca et al., 2013), the experimental C57BL/c mice were intraperitoneally infected with 1×10^6^ (100 μl) iRBCs from the donor mice. Tail blood was collected for thin smears and was stained with Giemsa dye four days after the PbA infection. Parasitemia was observed under a microscope.

### Sample Collection

The experimental mice were kept individually in cages. Fecal samples and intestinal contents were collected from 13 Ctrl mice and 13 PbA mice at 4 days post-infection (dpi) by scooping the feces into EP tubes with sterile tweezers. The intestine was snipped at the ileocecal junction for collection of the intestinal contents. Then, the feces and intestinal contents from the same mice were mixed to obtain enough samples for sequencing (Zhao et al., 2019) and immediately frozen in liquid nitrogen and stored at -80°C until use.

Samples packaged with 10 kg dry ice were sent to a company (Novogene Bioinformatics Technology Co., Ltd. in Tianjin, China), where they were stored at −80°C until DNA extraction.

### DNA Extraction, PCR Amplification, and Sequencing

DNA extraction of the fecal samples was performed by using the CTAB or SDS method. Then, PCR amplification and sequencing were carried out. Microbial 16S rRNA primers (16S V4: 515F-806R, 392 bp) were used. PCR was performed with the Phusion^®^ High-Fidelity PCR Master Mix (New England Biolabs). The PCR product mixture was purified with a Qiagen Gel Extraction Kit (Qiagen, Germany).

Sequencing libraries were generated using the TruSeq^®^ DNA PCR-Free Sample Preparation Kit (Illumina, USA) following the manufacturer’s recommendations, and index codes were added. The library quality was assessed on the Qubit@ 2.0 Fluorometer (Thermo Scientific) and Agilent Bioanalyzer 2100 system. Finally, the library was sequenced on an Illumina HiSeq 2500 platform, and 250 bp paired-end reads were generated. The raw data of all samples were uploaded into the Sequence Read Archive (SRA) in NCBI and registered with the BioProject database upon BioProject ID PRJNA719274 with BioSample accessions using a series of sequencing numbers from SAMN18593863 to SAMN18593888 (https://www.ncbi.nlm.nih.gov/sra/PRJNA719274).

### Data Processing and Statistical Analysis

Paired-end reads were assigned to the samples based on their unique barcode and truncated by cutting off the barcode and primer sequence. Paired-end reads were merged using FLASH V1.2.7 (Magoč and Salzberg, 2011). Quality filtering of the raw tags was performed with QIIME (V1.7.0) (Caporaso et al., 2010). The chimera sequences were detected using the UCHIME algorithm (Edgar et al., 2011) and were removed (Haas et al., 2011). Then, the effective tags were obtained. Sequence analysis was performed by Uparse software (Uparse v7.0.1001) (Edgar, 2013). Sequences with ≥97% similarity were assigned to the same OTUs. Representative sequences for each OTU were screened for further annotation. For each representative sequence, the GreenGene Database (DeSantis et al., 2006) was used based on the RDP 3 classifier (Version 2.2) (Wang et al., 2007) algorithm to annotate the taxonomic information. To study the phylogenetic relationships of the different OTUs and the differences among the dominant species in different samples (groups), multiple sequence alignment was conducted using MUSCLE software (Version 3.8.31) (Edgar, 2004).

OTU abundance information was normalized using a standard sequence number corresponding to the sample with the least sequences. Subsequent analyses of alpha diversity and beta diversity were all performed based on these normalized output data. Alpha diversity was applied to analyze the complexity of the species diversity for a sample through 6 indices, including Observed-species, Chao1, Shannon, Simpson, and Good-coverage. All of these indices in our samples were calculated with QIIME (Version 1.7.0) and displayed with R software (Version 2.15.3). Beta diversity analysis was used to evaluate the differences of the samples in terms of species complexity. Beta diversity of both weighted and unweighted UniFrac was calculated by QIIME software (Version 1.7.0). Cluster analysis was preceded by principal component analysis (PCA), which was applied to reduce the dimension of the original variables using the FactoMineR package and ggplot2 package in R software (Version 2.15.3). Principal coordinate analysis (PCoA) was performed to obtain the principal coordinates and visualize the complex, multidimensional data. A distance matrix of weighted or unweighted UniFrac among the samples was transformed to a new set of orthogonal axes, for which the maximum variation factor was demonstrated by the first principal coordinate and then the second maximum variation factor was demonstrated by the second principal coordinate, and so on. PCoA analysis was displayed by the WGCNA package, stat packages and ggplot2 package in R software (Version 2.15.3). The unweighted pair-group method with arithmetic means (UPGMA) clustering was performed as a type of hierarchical clustering method to interpret the distance matrix using the average linkage and was conducted with QIIME software (Version 1.7.0).

## Results

### General Information

**Figure 1:**
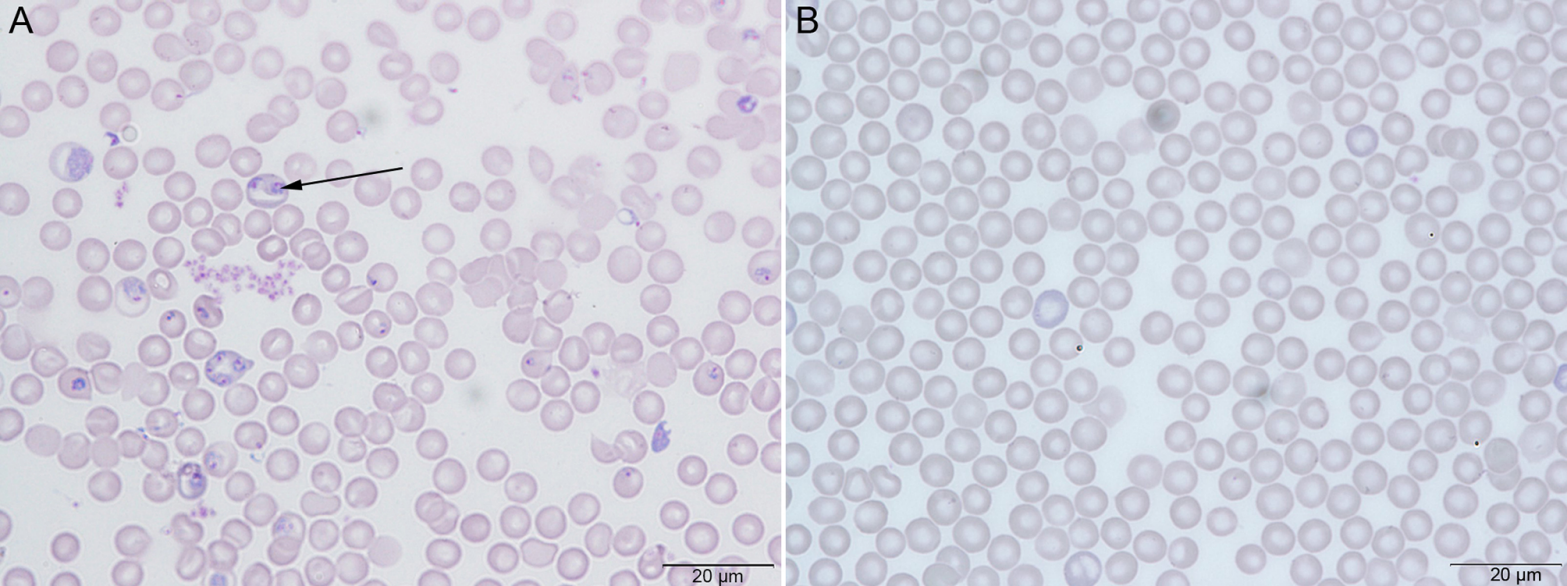
Giemsa’s stain to check the parasitemia of *Plasmodium berghei* ANKA (PbA) in C57BL/6 mice. **(A)** The PbA group with successful parasite infection. **(B)** The control group (Ctrl) without parasite infection.

On Day 0, mice in the PbA group were infected with PbA parasites. At the 4th dpi, parasites were observed in thin blood smears by microscopy, confirming successful infection (**Figure 1A**), while the Ctrl group was not infected (**Figure 1B**).

### Data and Sequencing

**Figure 2:**
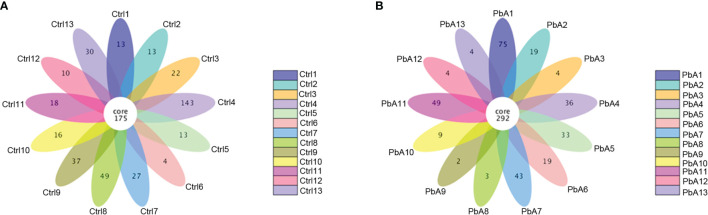
Operational taxonomic unit (OTU) analysis in the different fecal samples. **(A)** Flower diagram showing the shared and unique OTUs in the Ctrl samples. **(B)** Flower diagram showing the exclusive and mutual OTUs in the PbA samples.

A total of 26 fecal samples (13 PbA mice and 13 Ctrl mice) were collected and sent for sequencing. In total, 2,060,453 raw sequences were generated from all of the samples, and the number of sequences varied from 55,612 to 131,904. The mean number of sequences per sample was 79,248 ± 13,718 (standard deviation, SD). After quality filtering, clustering, and combining the PCR replicates, a total of 1,984,263 high-quality sequences remained, which ranged from 53,433 to 125,761 sequences per sample, with an average of 76,318 ± 13,047 (**Table 1**). The GC content in all samples was ranged from 51.51% to 54.51%, and the average was 53.47%. The high-quality reads were arranged with ≥ 97% sequence similarity into 15,367 microbial OTUs, which ranged from 418 to 752. Each sample had 591 OTUs, 533 observed species, and 76,318 sequences on average (**Table 1**). In general, the OTU number was increased after PbA infection compared with the Ctrl. **Table 1** lists the detailed data of each sample. A Venn diagram shows the similarities and differences between the communities in the two groups, while a flower diagram shows the similarities and differences between the communities in the different samples. There were a total of 932 OTUs in the two groups. There were 317 OTUs unique to the control mice, and 244 OTUs were unique to mice infected with PbA. The control mice had 73 more OTUs than the infected mice. For the individual samples, the maximum number of OTUs was 143 in Ctrl4, the minimum number of OTUs was 2 in PbA9, and the mean number of unique OTUs was 27 (**Figures 2A, B**).

**Table 1:** Operational taxonomic unit (OTU)-base diversity indices in infected and uninfected mouse gut samples.

Sample Name	Group	Raw PE (#)	Effective Tags (#)	Base (nt)	GC%	OTUs	Effective%	Observed_species	Shannon	Simpson	Chao1	ACE	Goods_coverage
Ctrl1	Ctrl	85,974	82,383	20,798,335	52.66	598	95.82	514	6.28	0.97	604.57	608.54	0.998
Ctrl2		80,902	77,369	19,530,139	54.29	617	95.63	535	6.52	0.97	569.04	583.38	0.999
Ctrl3		86,343	83,256	21,011,034	54.22	545	96.42	469	5.72	0.95	637.17	559.03	0.998
Ctrl4		131,904	125,761	31,725,297	54.51	698	95.34	589	6.18	0.97	682.79	697.71	0.998
Ctrl5		71,587	69,149	17,438,217	54.22	500	96.59	429	5.58	0.96	506.69	524.72	0.998
Ctrl6		73,247	70,695	17,830,968	54.04	418	96.52	353	5.28	0.95	415.26	415.45	0.999
Ctrl7		57,464	55,093	13,909,306	53.45	649	95.87	574	5.95	0.96	620.06	635.91	0.998
Ctrl8		72,763	69,880	17,655,849	52.66	671	96.04	574	5.17	0.91	631.01	656.52	0.998
Ctrl9		87,274	84,207	21,246,968	54.23	622	96.49	571	6.00	0.96	666.88	672.17	0.998
Ctrl10		78,469	76,083	19,193,698	54.47	530	96.96	467	5.58	0.96	495.50	524.30	0.999
Ctrl11		55,612	53,433	13,496,248	53.63	515	96.08	515	5.44	0.93	594.22	590.13	0.998
Ctrl12		76,039	74,580	18,845,774	52.22	526	96.37	478	3.98	0.71	533.38	546.06	0.998
Ctrl13		73,917	71,653	18,113,970	54.08	636	96.94	557	3.86	0.66	604.00	622.19	0.998
PbA1	PbA	86,302	83,224	21,023,038	53.47	725	96.43	657	6.52	0.97	741.43	742.38	0.998
PbA2		70,892	68,530	17,313,108	54.01	592	96.67	543	6.94	0.99	579.36	583.65	0.999
PbA3		78,122	75,372	19,027,335	53.24	499	96.48	467	5.47	0.93	515.36	530.14	0.998
PbA4		84,892	82,192	20,779,197	51.51	709	96.82	628	3.58	0.64	726.88	739.98	0.997
PbA5		85,930	83,028	20,959,257	53.61	734	96.62	682	6.38	0.97	797.16	763.79	0.998
PbA6		76,568	73,866	18,676,473	52.91	752	96.47	694	6.87	0.98	740.60	756.49	0.998
PbA7		78,238	74,674	18,879,497	53.43	648	95.44	604	6.99	0.98	649.63	660.18	0.998
PbA8		84,190	81,198	20,518,165	53.02	533	96.45	487	6.97	0.99	519.67	515.46	0.999
PbA9		80,595	77,260	19,534,882	52.08	444	95.86	403	3.47	0.71	432.22	448.84	0.999
PbA10		78,906	76,070	19,202,580	53.54	529	96.41	499	6.33	0.97	519.09	526.28	0.999
PbA11		62,271	59,508	15,033,763	53.27	639	95.56	585	6.50	0.97	628.67	632.08	0.999
PbA12		85,121	81,750	20,648,817	53.47	524	96.04	488	6.47	0.97	537.52	537.78	0.999
PbA13		76,931	74,049	18,698,056	53.95	514	96.25	491	7.02	0.99	521.36	522.34	0.999
Total		2,060,453	1,984,263	501,089,971	1390.19	15367	2502.57	13853	151.03	23.89	15469.50	15595.48	25.957
Maximum value		131,904	125,761	31,725,297	54.51	752	96.96	694	7.02	0.99	797.16	763.79	0.999
Minimum value		55,612	53,433	13,496,248	51.51	418	95.34	353	3.47	0.64	415.26	415.45	0.997
Average		79,248	76,318	19,272,691	53.47	591	96.25	533	5.81	0.92	594.98	599.83	0.998
SD		13,718	13,047	3,289,429	0.77	91.76	0.45	83.09	1.06	0.11	95.81	93.07	0.0006

Ctrl and PbA represent the uninfected (control) mouse group and the Plasmodium berghei ANKA-infected mouse group, respectively.

SD represents the standard deviation of the mean number.

The species accumulation boxplots showed that the species richness in all samples approached the equilibrium phase, which indicated that the number of species in the samples did not increase significantly with increasing sample size (**Supplementary Figure 1A**). Likewise, the rarefaction curves certified the species evenness in each sample had approached the saturation number level, and it showed that the sequencing data were reasonable, and more data would only produce a small number of new species (**Supplementary Figure 1B**).

### Taxonomic Overview

**Figure 3:**
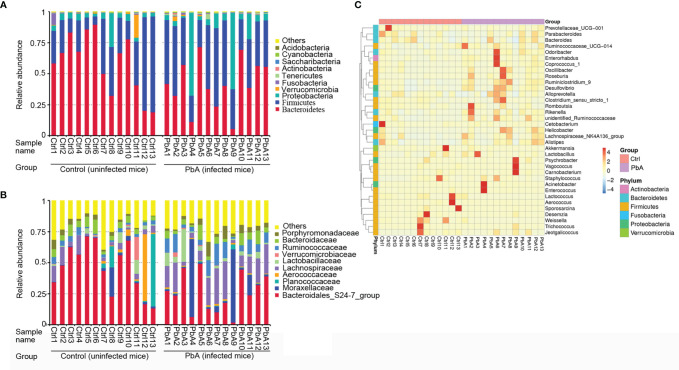
Changes in the microbial community components. Relative abundance of fecal bacterial phyla and families existing in C57BL/6 mice. Each bar represents the top 10 most abundant phyla and families, whereas “Other” was obtained from low abundance and unclassified OTUs. Sample names represent the samples described in **Table 1**. **(A)** Variations in the bacterial community components at the phylum level. **(B)** Variations in the bacterial community components at the family level. **(C)** Taxonomic heatmap of the two groups at the genus level. Typical relative abundance of diversified genera present in the Ctrl and PbA groups. Each column represents a unique subject.

**Figure 3** shows the bacterial community composition of the feces in the PbA and Ctrl mice. The cumulative column diagram of the species’ relative abundance was generated from the top 10 taxa, which comprised 97% of the reads.

At the phylum level (**Figure 3A**), *Bacteroidetes* (50.04%), *Firmicutes* (36.34%), *Proteobacteria* (10.65%), and *Verrucomicrobia* (1.11%) were the major components of the gut microbiota in all samples. *Bacteroidetes* and *Verrucomicrobia* were significantly decreased after PbA infection compared with Ctrl, while *Firmicutes* and *Proteobacteria* were considerably increased after PbA infection compared with the Ctrl. Obviously, *Proteobacteria* was substantially more abundant in the PbA group than in the Ctrl group. Conversely, *Bacteroidetes* was notably reduced in the PbA group compared with the Ctrl group.

At the family level (**Figure 3B**), the most dominant family was *Bacteroidales_S24–7_group* (35.83%) in all samples. *Lachnospiraceae* (10.84%), *Moraxellaceae* (7.35%), *Ruminococcaceae* (5.79%), *Bacteroidaceae* (5.27%), *Lactobacillaceae* (3.70%), *Porphyromonadaceae* (3.63%), *Planococcaceae* (3.53%), *Aerococcaceae* (3.03%), and *Verrucomicrobiaceae* (1.11%) were the subdominant families. *Lachnospiraceae* and *Moraxellaceae* were significantly increased after PbA infection compared with Ctrl, while *Bacteroidales_S24–7_group*, *Planococcaceae*, and *Verrucomicrobiaceae* were vastly decreased after PbA infection compared with Ctrl. Obviously, *Bacteroidales_S24-7_group* was reduced from 45.93% to 25.73%. At the genus level, the significant differences between the two groups were seen in several taxa. In **Figure 3C**, the most representative genus is displayed for each group.

### Analysis of the Bacterial Community Within Groups

Alpha diversity was used to analyze the microbiota community diversity in the samples (Within-community), and the diversity analysis (Alpha diversity) of a single sample could reflect the richness and diversity of the microbiota community in the sample. The alpha diversity index, mainly including the number of observed OTUs, goods coverage, Chao1, Shannon and Simpson diversity index, and phylogenetic diversity (**Table 1**), manifested a trend that PbA infection transiently changed the richness and evenness. Notable differences were found in the observed and estimated OTUs between the PbA and Ctrl groups. Observed species, Shannon index values, and Simpson index values were added to the PbA group compared with the Ctrl group. At the same time, the OTU number was reduced in the PbA group compared with the Ctrl group (**Supplementary Figure 2**).

For the Chao1 analysis (**Supplementary Figure 2A**), Ctrl samples had more estimated OTUs than PbA (*p* = 0.488, t-test; *p* = 0.6498, Wilcoxon rank-sum test). For the observed species analysis (**Supplementary Figure 2B**), the PbA group samples had a higher number than the Ctrl group samples (*p* = 0.1597, t-test; *p* = 0.228, Wilcoxon rank-sum test). For Shannon diversity (**Supplementary Figure 2C**), samples in the PbA group also had a higher number than those in the Ctrl group (*p* = 0.1464, t-test; *p* = 0.01831, Wilcoxon rank-sum test). Outliers were detected in both the Ctrl and PbA groups for Shannon diversity. For phylogenetic diversity (**Supplementary Figure 2D**), samples in the PbA group had a slightly higher number than those of Ctrl group (*p* = 0.6456, t-test; *p* = 0.9598, Wilcoxon rank-sum test).

### Analysis of the Bacterial Community Between the Groups

For unweighted UniFrac (**Supplementary Figure 2E**) concerning the presence or absence of changes in the species, the beta diversity of the gut samples was different between the Ctrl and PbA groups. The values were decreased in the Ctrl relative to PbA group. The PbA group samples had a higher number of OTUs than the Ctrl group (*p* = 0.0002, t-test; *p* = 0.0012, Wilcoxon rank-sum test). It also revealed that the discreteness of the beta diversity increased from Ctrl to PbA. However, for the weighted UniFrac analysis concerning both the presence or absence of species and the changes in species abundance, the values were similar between the Ctrl and PbA groups (**Supplementary Figure 2F**).

**Figure 4:**
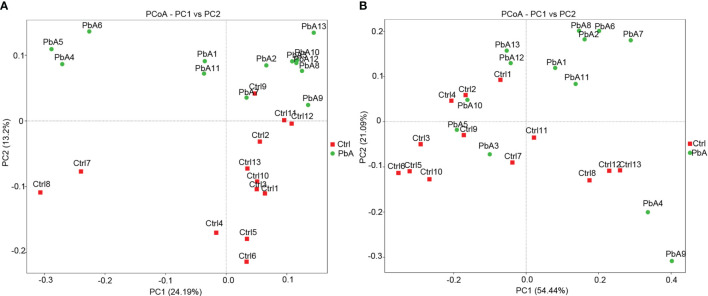
Principal coordinates analysis (PCoA) based on unweighted **(A)** and weighted **(B)** UniFrac distance matrixes of fecal samples of C57BL/6 mice during infection. The fecal bacterial community components of both Ctrl- and PbA- infected mice are represented. Sample names are as described in **Table 1**.

The similarity degree between the microbiota composition of each sample was tested by using the PCoA (**Figure 4**) based on unweighted (**Figure 4A**) and weighted (**Figure 4B**) UniFrac distance matrixes. The gut microbiota changed markedly as the PbA infection progressed in the C57BL/6 mice. The similarity degree between community structures manifested by PCoA was examined by comparing within-group unweighted UniFrac distances for PC2 axis between the Ctrl and PbA. On the PCoA plot, each symbol represents the gut microbiota of one mouse. The microbiota of the PbA-infected feces was distinctly different from those of the Ctrl feces (**Figure 4A**). The relationships between the community structures revealed by PCoA were further examined by comparing the within-group weighted UniFrac distances (**Figure 4B**).

In accordance with the PCoA plot, the within-group distances were considerably lower than the between-group distances for each group (ANOSIM, MRPP, *p* ≤ 0.01). The R-value of the among-group dissimilarities analysis *via* analysis of similarities (ANOSIM, **Supplementary Table 1**) was 0.1689, which indicated remarkable differences between the PbA and Ctrl groups. The P-value of the ANOSIM was 0.002, which manifested statistical significance (*p* < 0.05). Among-group dissimilarities analysis *via* multi-response permutation procedure (MRPP, 0.5658, 0.5889; observed-delta, expected-delta) revealed a lower difference within the group and a higher difference between the groups. The A-value of the MRPP (**Supplementary Table 2**) was 0.03928, which demonstrated that the differences between groups were higher than those within groups (A > 0). Significance < 0.05 made clear noteworthy differences. These data suggested that the microbiota composition of PbA feces was remarkably different from those of Ctrl feces. Additionally, these 26 samples were clustered by UPGMA with a weighted UniFrac matrix. The main samples of the Ctrl and PbA groups were generally clustered into two individual clusters at the phylum level (**Supplementary Figure 3**).

### Potential Biomarkers Discovery

**Figure 5:**
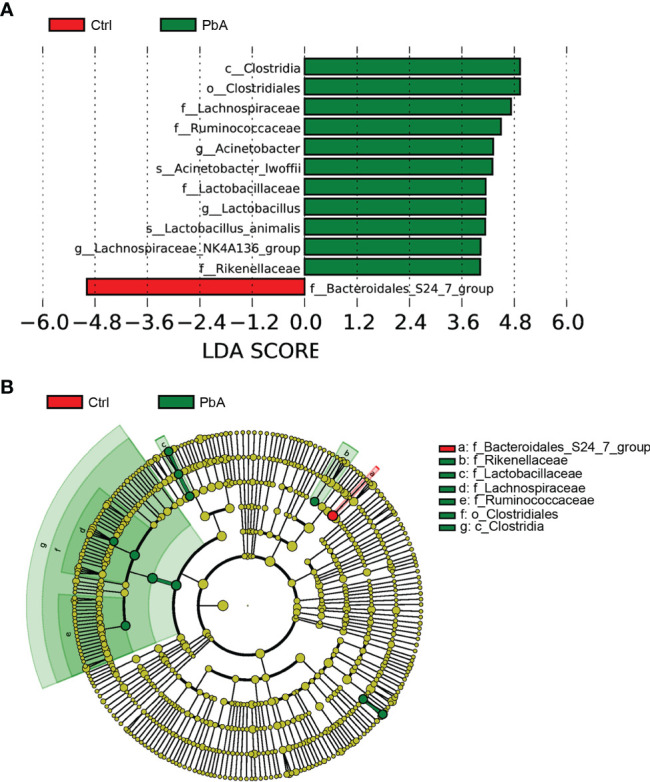
The most differential abundant taxa in different groups. **(A)** LEfSe identified the bacterial taxa that significantly differentiated the Ctrl and PbA mice. Histogram of the linear discriminant analysis (LDA) scores distribution between the Ctrl and PbA groups. Only taxa with statistical differences between groups are displayed in the figure, which was determined by meeting an LDA significance threshold > 4. **(B)** Cladogram of the most differentially abundant bacterial taxa in the two groups. The red and green colors represent the bacterial communities of the Ctrl and PbA groups, respectively.

LEfSe analysis identified the different statistical biomarkers, which were the bacterial taxa with significant differences between the Ctrl and PbA groups. Therefore, we conducted LEfSe on the top 10 taxa (average relative abundance > 0.0001, **Figure 5A**). This threshold (LDA Score > 4) could obtain as many taxa as possible for meaningful comparisons in the analysis. The potential biomarkers at different taxonomic levels were determined in the PbA and Ctrl groups (**Figure 5B**). At the genus level, the biomarkers with a remarkable difference between the PbA and Ctrl groups were *Acinetobacter*, *Lactobacillus*, and *Lachnospiraceae_NK4A136_group*.

To generate the OTU co-abundance units, a method similar to gene coexpression network construction was used (**Supplementary Figure 4**). These results showed that an OTU co-abundance network method could successfully generate associations that could recapitulate the useful data, and therefore, it might have potential value for identifying microbial associations. Combined with LEfSe for the top 10 taxa and networks, they showed that the genus *Acinetobacter* was correlated with the genera *Aerococcus*, *Christensenellaceae_R_7_group*, *Psychrobacter*, and *Proteus*. The genus *Lactobacillus* was correlated with the genera *Streptococcus*, *Erysipelatoclostridium*, *Enterorhabdus*, *Family_XIII_AD3011_group*, *Ruminocococcaceae_UCG_014*, *Eubacterium_ruminantium_group*, and so on. The genus *Lachnospiraceae_NK4A136_group* was correlated with many genera.

## Discussion

Malaria is a prevalent infectious disease worldwide, especially in tropical developing regions (Sugar et al., 2017). Malaria co-infection with bacteria may cause enteritis, urinary tract infection, meningitis, pneumonia, sepsis, and sinusitis, but research concerning the impact of parasites *Plasmodium* spp. on the gut microbiota of mice is limited. Although studies have demonstrated that PbA causes gut microbiota alterations, the potential biomarkers correlated with ECM’s pathogenesis remain unclear.

In this study, the gut microbiota in mice concerning their correlation with PbA infection was studied. In other words, the gut microbiota compositions in PbA-infected and uninfected C57BL/6 mice were studied. Both species accumulation boxplots and rarefaction curves approximated the saturation level, which indicated nearly complete coverage of the total microbiota diversity. Based on the alpha and beta diversity analysis, we found that the diversity and richness of the gut microbiota in all fecal samples were changed after PbA infection. In the current study, the gut microbiota at the phylum level in both uninfected and infected mice was dominated by *Firmicutes*, *Bacteroidetes*, and *Proteobacteria*, which were consistent with the previous study (Taniguchi et al., 2015; Fan et al., 2019).

The present data also show that the relative richness of dominant phyla and families changed after infection. PbA infection decreased the richness of *Bacteroidetes* and *Verrucomicrobia* in the fecal samples compared with Ctrl. Conversely, PbA infection increased the *Firmicutes* and *Proteobacteria* in the feces compared with Ctrl. These results were different from those of a previous study (Taniguchi et al., 2015). A previous study demonstrated that *Bacteroidetes* in the gut was correlated with metabolic disease (Johnson et al., 2017). A review showed that *Proteobacteria* could cause metabolic disorders and inflammatory bowel disease (Rizzatti and Lopetuso, 2017). These changes showed that the metabolism of mice was altered after PbA infection. In addition, *Bacteroidales_S24–7_group* was the most dominant family in all samples, while *Bacteroidales_S24–7_group* clearly decreased after PbA infection compared with Ctrl. A previous study manifested that antidepressant drugs altered the composition levels of *Bacteroidales* in the gut microbiota in depressive susceptible mice, which could ultimately suggest ways to treat depression disease (Qu et al., 2017). The diversity in the gut decreased after PbA infection, which showed that the changes in gut microbiota composition correlated with nervous system disease. C57BL/6 mice with PbA infection share similar symptoms to humans infected with *P. falciparum*, indicating it is an appropriate model of ECM. Infection with PbA parasites altered the gut microbiota composition in C57BL/6 mice. That is, HCM caused by *P. falciparum* might be associated with alterations in the gut microbiota. Consistent with this notion, a study revealed a remarkable association between the host microbiota composition and *P. falciparum* infection (Yooseph et al., 2015; Waide et al., 2020).

At the same time, the families *Lachnospiraceae* and *Moraxellaceae* were significantly increased after PbA infection compared with Ctrl, while the families *Planococcaceae* and *Verrucomicrobiaceae* were decreased. These results are not consistent with the findings by Taniguchi et al., 2015. A previous study showed that the abundance of the family *Lachnospiraceae* was correlated with most modified metabolites. For example, a reduction in short-chain fatty acid (SCFA)-producing bacteria influenced the shape of the metabolomics profile, playing a role in several metabolites. Bacteria in the family *Moraxellaceae* were found to be distinctly correlated with laryngotracheal stenosis (Hillel et al., 2019). An increase of the family *Moraxellaceae* showed that the mice might have respiratory symptoms after PbA infection. Several subdominant phyla or families, such as *Verrucomicrobiaceae*, should be given some attention. *Verrucomicrobia* is common bacteria in soil, and it is also found in the ocean (Freitas et al., 2012). However, its role in the rodent gut is poorly understood.

In this study, LEfSe analysis was conducted to identify the biomarkers with significant differences between the Ctrl and PbA groups. Some bacterial genera, such as *Acinetobacter*, *Lactobacillus*, and *Lachnospiraceae_NK4A136_group*, were overrepresented in the PbA*-*infected fecal microbiota. Consistent with this notion, a previous study revealed that the increased abundances of *Lactobacillus* and *Bifidobacterium* were associated with the severity of malaria (Villarino et al., 2016). A significant species of the genus *Acinetobacter* (species *Acinetobacter calcoaceticus*) was associated with the nosogenesis of multiple sclerosis which was an autoimmune disease. In addition, this species can induce proinflammatory responses for mononuclear cells in human peripheral blood (Cekanaviciute et al., 2017). *Lactobacillus* was demonstrated to play a role in sleep and stress responses in patients (Hemarajata and Versalovic, 2013; Aizawa et al., 2018). A previous study demonstrated that suppressing gut inflammation or utilizing resistant competitors (probiotics) might be potential methods to limit the reproduction of pathogenic bacteria (Pickard et al., 2017). At the same time, a method similar to gene coexpression network construction was used to generate the OTU co-abundance units. Combined with LEfSe for the top 10 taxa and networks, they showed that the genus *Acinetobacter* was correlated with the genera *Aerococcus*, *Christensenellaceae_R_7_group*, *Psychrobacter*, and *Proteus*. The genus *Lactobacillus* was correlated with the genera *Streptococcus*, *Erysipelatoclostridium*, *Enterorhabdus*, *Family_XIII_AD3011_group*, *Ruminocococcaceae_UCG_014*, *Eubacterium_ruminantium_group*, and so on. The genus *Lachnospiraceae_NK4A136_group* was correlated with many genera. Cattoir et al. discovered that species belonging to the genus *Aerococcus* were related to patients suffering from urinary tract infections (Cattoir et al., 2010). Zhou et al. revealed that the genus *Enterorhabdus* was involved in the pathologic development of AD and other central nervous system diseases (Zhou et al., 2019). The *Family_XIII_AD3011_group* was correlated with several polycystic ovary syndrome-related markers (Lüll et al., 2021). Shimizu et al. demonstrated that the genera *Christensenellaceae*_R-7_group and *Ruminococcaceae_UCG-010* had positive correlations with obesity (Shimizu et al., 2021). These findings offered more insights into host intestinal microbiota variation after PbA infection and demonstrated that microbiota analysis could play a meaningful role in the early diagnosis of curable malaria and the perception of the pathogenesis of malaria.

Taniguchi et al. used C57BL/6 and BALB/c mice infected with PbA to show that changes in the gut microbiota were related to intestinal pathological changes (Taniguchi et al., 2015). The number of mice per group was only five in their study, while the number of mice per group was higher in our study. Fan et al. found that the gut microbiota composition in C57BL/6 mice was reconstructed after infection by the elimination of blood-stage PbA (Fan et al., 2019). However, the gut microbiota composition was only studied for many OTUs instead of specific bacterial names.

Although this research studied the alteration of the gut microbiota profile in C57BL/6 mice induced by PbA infection and discovered potential biomarkers correlated with experimental cerebral malaria, the absence of direct evidence indicates there will be challenges in studying the mechanisms correlated with the observed changes in the gut microbiota. This study also had some limitations. In future studies, it will be better to study changes that occur at several days after infection, such as 0, 3, 5, and 7 dpi. Next, the pathogenesis of malaria needs to be profoundly studied correlated with the metabolism associated with the changed gut microbiota in the mice infected with PbA. In addition, the fresh feces or intestinal contents recommended by the company for sequencing were at least more than 0.5 grams at that time. In order to guarantee the sufficient sample quality and data quality, we collected twice the sample quantity as company’s recommendation. And the infected mice were in very poor physical condition, thus it caused them to eat less and produce less feces. Thus, the mixed samples of feces and intestinal content were prepared. In future studies, as sequencing technology improves, it will be better to collect the fresh feces or the intestinal contents separately to study their microbiota composition.

In summary, the gut microbiota in PbA*-*infected and uninfected mice was characterized. Bacterial taxa overrepresented in the infected and uninfected groups were identified. At the same time, some potential biomarkers at different taxonomic levels were obtained. These microbial taxa can serve as direct targets to clarify their roles in the pathogenesis and progression of cerebral malaria in future studies. Additionally, in the association study of gut microbiota composition and PbA infection risk, strategic modulation of gut microbiota composition might decrease the PbA infection risk and possibly serve as a standard for some antimalarial drugs or malaria vaccines.

## Data Availability Statement

The datasets presented in this study can be found in online repositories. The names of the repository/repositories and accession number(s) can be found below: https://www.ncbi.nlm.nih.gov/bioproject/PRJNA719274/.

## Ethics Statement

The animal study was reviewed and approved by Institutional Animal Care and Use Committee of the Hubei University of Medicine under permit number HBMU-S20160414 and performed in the Collegial Laboratory Animal Center.

## Author Contributions

WG, SY, YZ, YX, and JL conceived the study and participated in its design. WG, SY, YY, and JL carried out the experiments. WG and JL performed the data analysis and interpretation, and drafted the manuscript. WC, XS, and WD participated in analyzing and interpreting the data. All authors contributed to the article and approved the submitted version.

## Funding

The Initial Project supported this study for Post-Graduates of Hubei University of Medicine (Grant No. 2016QDJZR04), the Research Project of Hubei Provincial Department of Education (Grant No. Q20172102), and the Principle Investigator Program of Hubei University of Medicine (Grant No. HBMUPI202101).

## Conflict of Interest

The authors declare that the research was conducted in the absence of any commercial or financial relationships that could be construed as a potential conflict of interest.

## Publisher’s Note

All claims expressed in this article are solely those of the authors and do not necessarily represent those of their affiliated organizations, or those of the publisher, the editors and the reviewers. Any product that may be evaluated in this article, or claim that may be made by its manufacturer, is not guaranteed or endorsed by the publisher.

## Supplementary Material

Supplementary Figure 1 Species richness and diversity analysis of mouse fecal samples after inoculation. **(A)** Species accumulation boxplots. **(B)** Rarefaction curves.

Supplementary Figure 2 Bacterial community comparison of the Ctrl group and PbA group. Outliers are marked as single points. **(A)** Alpha diversity analysis based on the Chao1 index between the two groups; **(B)** Alpha diversity analysis based on the observed_species index between the two groups; **(C)** Alpha diversity analysis based on the Shannon index between the two groups; **(D)** Alpha diversity analysis based on the PD_whole_tree index between the two groups; **(E)** Beta diversity analysis based on unweighted UniFrac; **(F)** Beta diversity analysis based on weighted UniFrac.

Supplementary Figure 3 Clustering tree of fecal samples by UPGMA according to their weighted UniFrac matrix. The left figure shows the UPGMA cluster tree structure, and the bars in the right figure show the relative abundance of the main bacterial groups at the phylum level. Phyla representing less than 1% of the sequences in a group were grouped as others.

Supplementary Figure 4 OTU co-abundance network showing modules of OTUs at the genus level. Different nodes represent different genera, and node size represents the average relative abundance of the genus, nodes of the same phylum have the same color, the thickness of the connecting line between the nodes is positively correlated with the absolute value of the correlation coefficient of species interaction, and the color of the connecting line is positively correlated with the correlation (Red is positively correlated, blue is negatively correlated).

Supplementary Table 1 Within- versus Among-Group Dissimilarities analysis *via* Analysis of Similarities (ANOSIM). R-value is between (-1, 1), and when the R-value is greater than 0, it indicates significant differences between groups. The credibility of the statistical analysis is expressed by the P-value, and P<0.05 indicates statistical significance. Significance < 0.05 indicates a significant difference.

Supplementary Table 2 Within- versus Among-Group Dissimilarities analysis *via* the Multi Response Permutation Procedure (MRPP). A smaller value of the observed delta indicates that the difference within the group is lower; a larger value of the expected delta indicates that the difference between the groups is higher; A > 0 demonstrates that the difference between the groups is higher than that within the groups; A < 0 indicates that the difference within the groups is higher than that between the groups. P < 0.05 indicates a significant difference.
