# Supplementary figures and images for: Observation of the Gut Microbiota Profile in C57BL/6 Mice Induced by Plasmodium berghei ANKA Infection
Source: Front Cell Infect Microbiol. 2021 Oct 28;11:680383. doi: 10.3389/fcimb.2021.680383 (PMC8581563; doi:10.3389/fcimb.2021.680383)

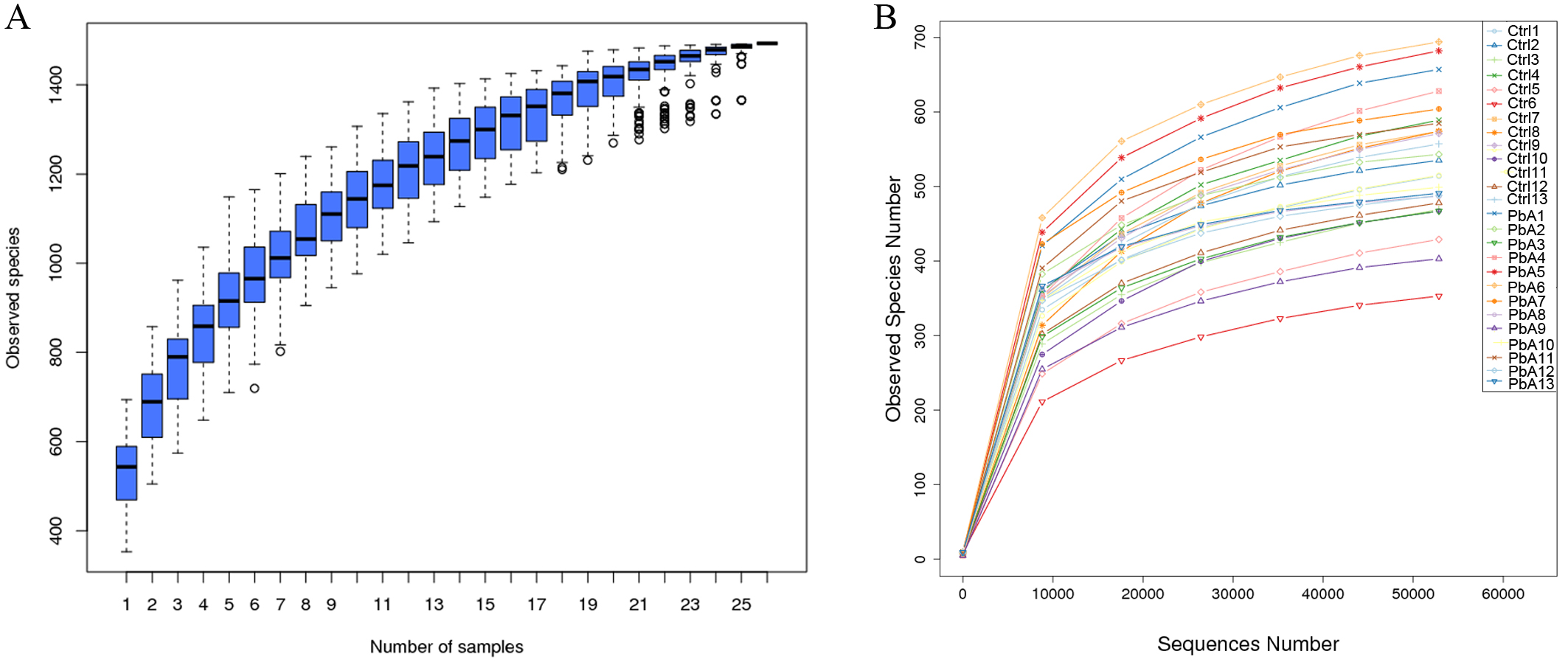

Supplement: Supplementary Figure 1 — Species richness and diversity analysis of mouse fecal samples after inoculation. (A) Species accumulation boxplots. (B) Rarefaction curves. [file Image_1.jpeg]

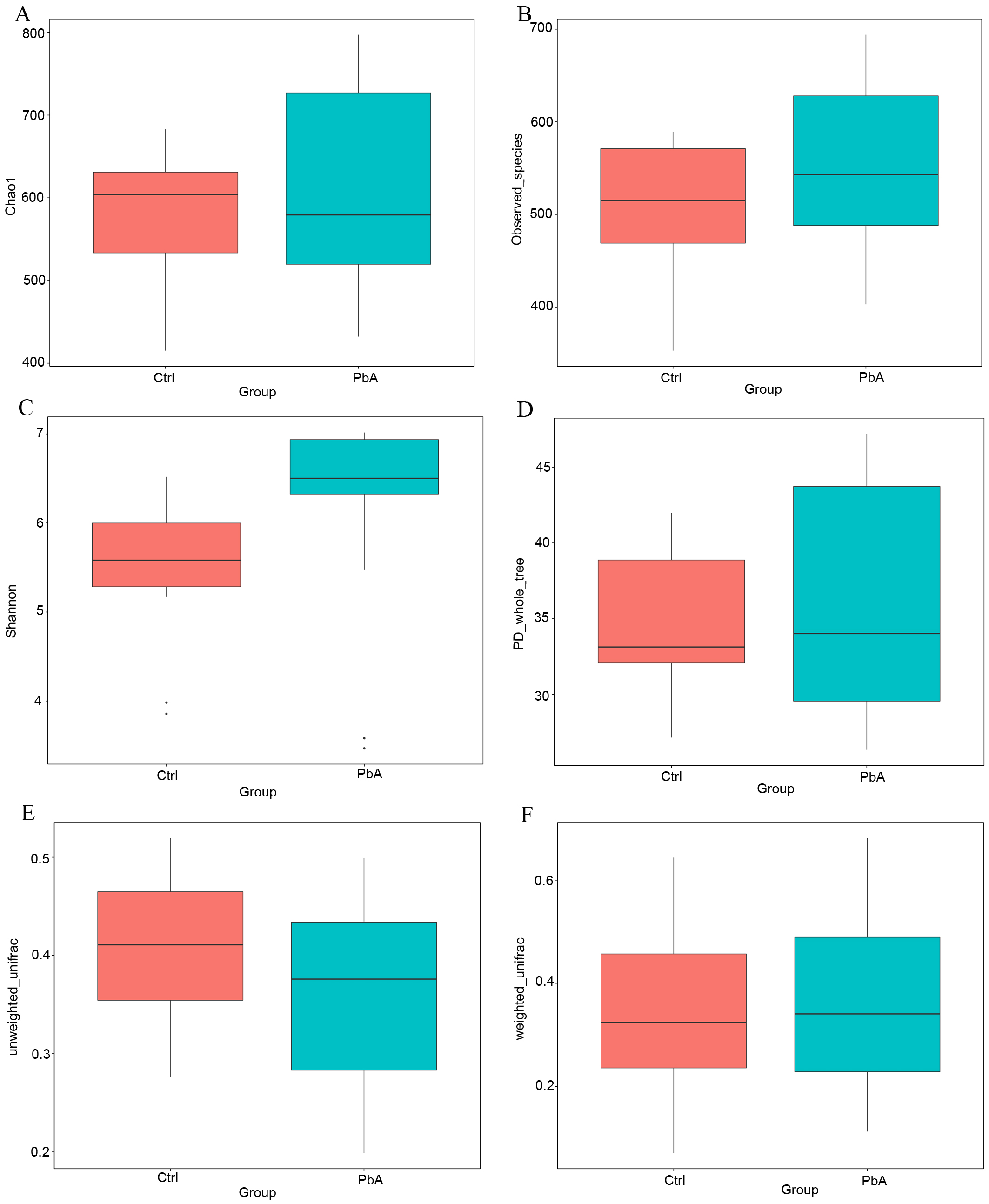

Supplement: Supplementary Figure 2 — Bacterial community comparison of the Ctrl group and PbA group. Outliers are marked as single points. (A) Alpha diversity analysis based on the Chao1 index between the two groups; (B) Alpha diversity analysis based on the observed_species index between the two groups; (C) Alpha diversity analysis based on the Shannon index between the two groups; (D) Alpha diversity analysis based on the PD_whole_tree index between the two groups; (E) Beta diversity analysis based on unweighted UniFrac; (F) Beta diversity analysis based on weighted UniFrac. [file Image_2.jpeg]

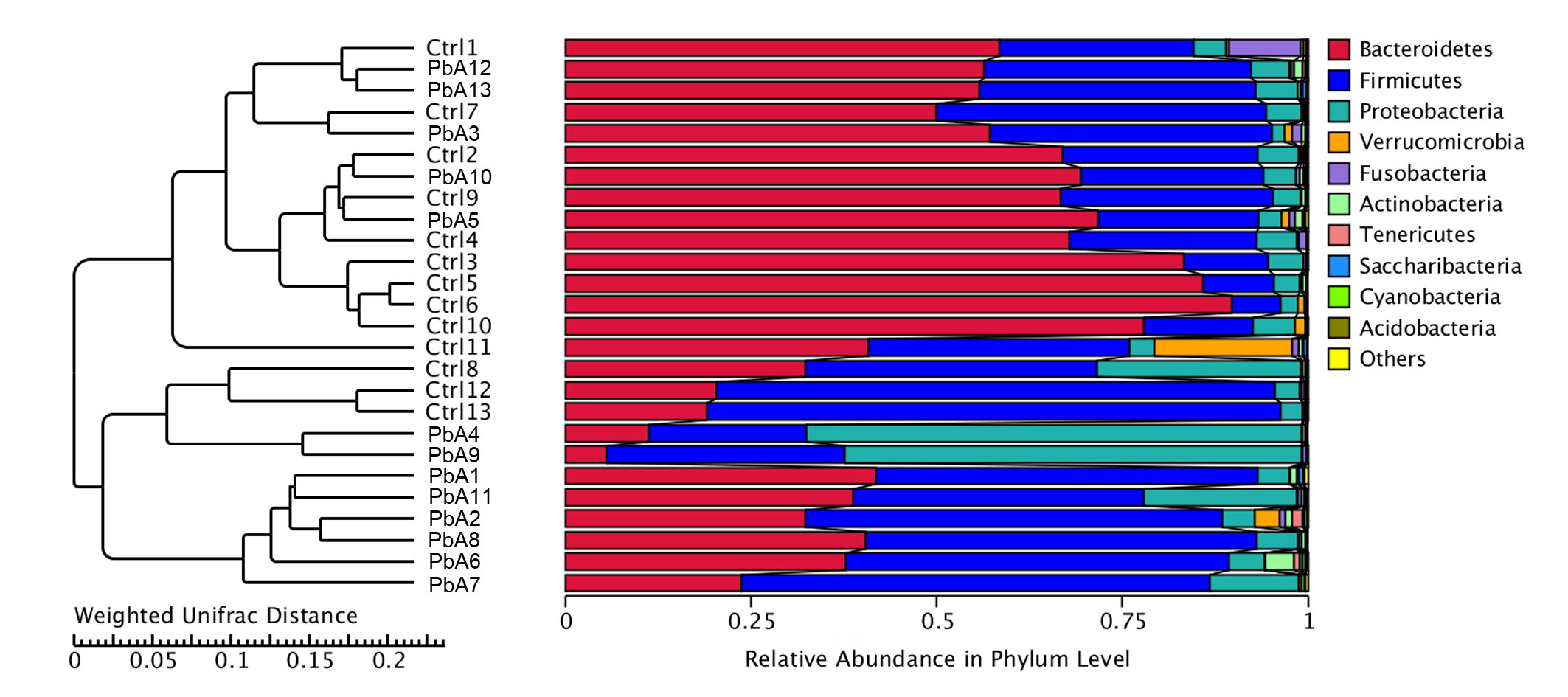

Supplement: Supplementary Figure 3 — Clustering tree of fecal samples by UPGMA according to their weighted UniFrac matrix. The left figure shows the UPGMA cluster tree structure, and the bars in the right figure show the relative abundance of the main bacterial groups at the phylum level. Phyla representing less than 1% of the sequences in a group were grouped as others. [file Image_3.jpeg]

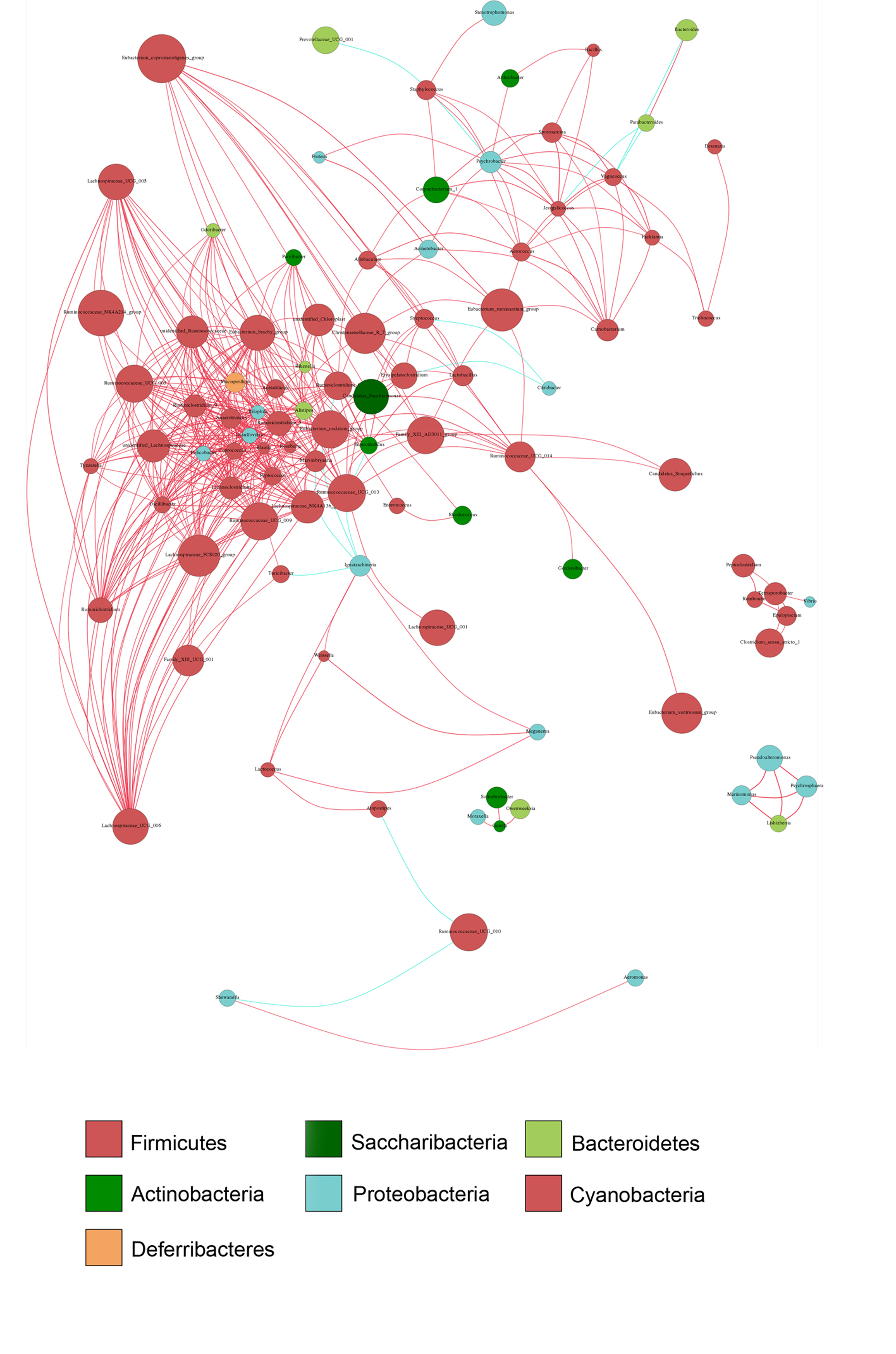

Supplement: Supplementary Figure 4 — OTU co-abundance network showing modules of OTUs at the genus level. Different nodes represent different genera, and node size represents the average relative abundance of the genus, nodes of the same phylum have the same color, the thickness of the connecting line between the nodes is positively correlated with the absolute value of the correlation coefficient of species interaction, and the color of the connecting line is positively correlated with the correlation (Red is positively correlated, blue is negatively correlated). [file Image_4.jpeg]
